# Zika Virus and Neuropathogenesis: The Unanswered Question of Which Strain Is More Prone to Causing Microcephaly and Other Neurological Defects

**DOI:** 10.3389/fncel.2021.695106

**Published:** 2021-09-30

**Authors:** Emily Louise King, Nerea Irigoyen

**Affiliations:** Division of Virology, Department of Pathology, University of Cambridge, Cambridge, United Kingdom

**Keywords:** Zika virus, viral lineages, neuropathogenesis, neural progenitor cells, apoptosis, unfolded protein response, premature differentiation

## Abstract

Despite being perceived to be a relatively innocuous pathogen during its circulation in Africa in the 20th century, consequent outbreaks in French Polynesia and Latin America revealed the Zika virus (ZIKV) to be capable of causing severe neurological defects. Foetuses infected with the virus during pregnancy developed a range of pathologies including microcephaly, cerebral calcifications and macular scarring. These are now collectively known as Congenital Zika syndrome (CZS). It has been established that the neuropathogenesis of ZIKV results from infection of neural progenitor cells in the developing cerebral cortex. Following this, two main hypotheses have emerged: the virus causes either apoptosis or premature differentiation of neural progenitor cells, reducing the final number of mature neurons in the cerebral cortex. This review describes the cellular processes which could potentially cause virus induced apoptosis or premature differentiation, leading to speculation that a combination of the two may be responsible for the pathologies associated with ZIKV. The review also discusses which specific lineages of the ZIKV can employ these mechanisms. It has been unclear in the past whether the virus evolved its neurotropic capability following circulation in Africa, or if the virus has always caused microcephaly but public health surveillance in Africa had failed to detect it. Understanding the true neuropathogenesis of ZIKV is key to being prepared for further outbreaks in the future, and it will also provide insight into how neurotropic viruses can cause profound and life-long neurological defects.

## Introduction

The Zika virus (ZIKV) was first identified in a febrile monkey in Uganda in 1947, and consequently circulated through the African continent, causing either an asymptomatic or self-resolving influenza like illness. However, outbreaks in French Polynesia (2013–2014) and South America (2015–2016) revealed that the virus could cause serious neurological health conditions. The French Polynesian epidemic coincided with a significant increase in the occurrence of Guillain-Barré syndrome in adults, a polyneuropathy mediated by the immune system that can lead to paralysis and death (Dirlikov et al., 2018). In addition, Brazil recorded a 20-fold increase in the incidence of microcephaly in newborns, which is characterised by a reduced head circumference of at least two standard deviations below the mean for those of the same gestational age and sex (DeSilva et al., 2017). The correlation of these cases with the identification of ZIKV-RNA in amniotic fluid led to the conclusion that ZIKV had teratogenic effects (Brasil et al., 2016). Consequently, in early 2016, ZIKV was declared by the WHO as a Public Health Emergency of International Concern (PHEIC; WHO, 2016). Questions were raised following the South American Zika epidemic: what made the current American strain so neuropathogenic in comparison to its ancestral African lineage?

This review discusses the pathological mechanisms that cause microcephaly following infection with the American ZIKV strain and addresses the possibility that these can also be employed by other ZIKV strains, leading to speculation that ZIKV might have been causing microcephaly in Africa for many years. Understanding the neuropathogenicity of the American ZIKV strain is key to being prepared for ZIKV outbreaks in the future.

### Genome and Life Cycle

**FIGURE 1:**
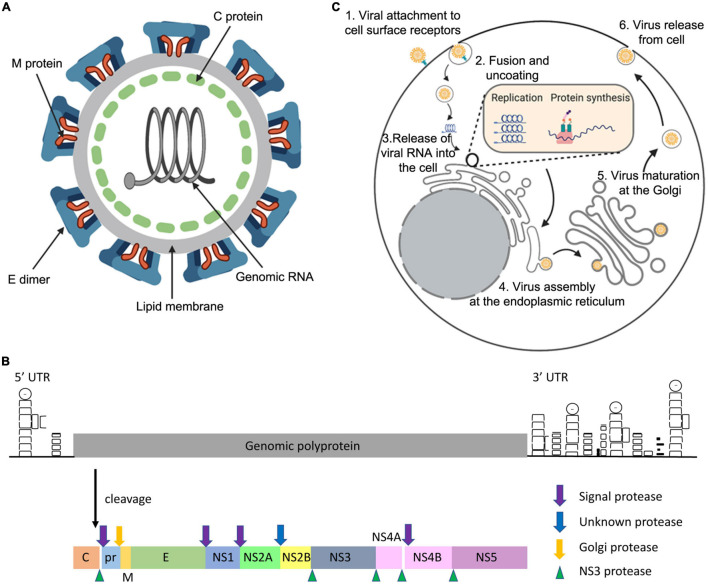
General features of the ZIKV. The ZIKV virion **(A)** is an enveloped, spherical virus about 50 nm in diameter. The surface proteins envelope (E) and membrane (M) are arranged in an icosahedral structure that encases the nucleocapsid **(C)** protein bound to the viral RNA (adapted from viralzone.com and created with Biorender.com). The genome **(B)** is positive sense single-stranded RNA consisting of a single open reading frame, and 5′ and 3′ untranslated regions. The single synthesised polyprotein is post-translationally cleaved by host proteases (signal and Golgi peptidases) and the viral NS3 protease. This forms the three structural proteins: C, pre-membrane (prM), and E. Additionally, there are seven non-structural proteins which are necessary for genome replication and the modification of host cellular functions (adapted from Pierson and Diamond, 2018). **(C)** ZIKV enters the host cell using receptor mediated endocytosis via the receptors DC-SIGN, Tyrosine 3, TIM-1 or ACL. ZIKV is then translocated via endosome vesicles to the ER, and viral RNA is released. Translation occurs and the resulting polyprotein is subsequently cleaved using host and viral proteases. ZIKV remodels the ER and uses viral replication factories to carry out genome replication. Immature virions then assemble on the ER derived membrane, undergo maturation at the Golgi, and are released from the cell using the secretory pathway (adapted from Gerold et al., 2017, created with Biorender.com).

ZIKV is a member of the genus *Flavivirus*. Mature ZIKV virions contain 180 copies of the envelope (E) and membrane (M) proteins, displaying an icosahedral shell of 90 flat-lying E dimers that encase the capsid (C) and the viral genome (Figure 1A; Tan et al., 2020). The genome is positive sense single-stranded RNA consisting of a single open reading frame, and 5′ and 3′ untranslated regions. The single synthesised polyprotein is post-translationally cleaved by host and viral proteases into three structural and seven non-structural (NS) proteins (Figure 1B; Pierson and Diamond, 2018).

Interaction of ZIKV with the host cell receptors DC-SIGN, Tyrosine 3, TIM-1 or ACL facilitates host cell entry via receptor mediated endocytosis (Hamel et al., 2015). Following protein synthesis and cleavage, ZIKV remodels the endoplasmic reticulum (ER) to form viral replication factories in order to replicate the genome (Cortese et al., 2017). Immature non-infectious virions assemble on the ER derived membrane and subsequently migrate through the secretory pathway.

Exposure to the mildly acidic Golgi environment causes a conformational change and cleavage of the M protein precursor (prM) by a host furin-like protease to form mature virions, which are consequently released from the cell (Figure 1C; Pierson and Diamond, 2018).

### Transmission

The most prominent mechanism of ZIKV transmission is via mosquitoes of the genus *Aedes*, particularly *Aedes aegypti* and *Aedes albopictus* (Musso et al., 2014). Following a bite from a ZIKV infected female mosquito, the virus is able to replicate in keratinocytes and dendritic cells, before dissemination via lymph nodes and the bloodstream (Alves et al., 2018; Boyer et al., 2018). However, it has also been shown that ZIKV is capable of transmission via sexual contact, most commonly from males to females. This is possible as ZIKV exhibits a cellular tropism for Sertoli cells of the testis, where the virus replicates and forms a reservoir (Pierson and Diamond, 2018). The virus can consequently persist there for months following viral clearance from the blood (Nicastri et al., 2016; Kurscheidt et al., 2019).

Sexual transmission of ZIKV has been studied using a mouse model. Clancy et al. (2018) showed that reproductive fluid derived from the epididymis and *vas deferens* (structures responsible for sperm transport from the testes) and from the prostate and seminal vesicle (accessory sex glands) contained ZIKV RNA when the mouse had previously been subcutaneously inoculated with ZIKV. Moreover, intravaginal inoculation of a female mouse with either of these fluids caused infection, evidenced by the presence of ZIKV RNA in the blood (Clancy et al., 2018).

Furthermore, the dramatic increase in identified microcephaly cases in Brazil during the 2015 Zika epidemic led to conclusions that the virus can undergo vertical transmission from mother to foetus (Musso et al., 2019). This was confirmed upon finding the presence of ZIKV RNA in the cerebral tissue of foetuses diagnosed with microcephaly, in addition to the maternal amniotic fluid (Zhang et al., 2016). Whilst this has the potential to occur in all trimesters of pregnancy, association of vertical transmission with “Congenital Zika syndrome” (CZS) is much stronger in the first trimester (Brasil et al., 2016; Musso et al., 2019).

### Lineage and Global Spread

**FIGURE 2:**
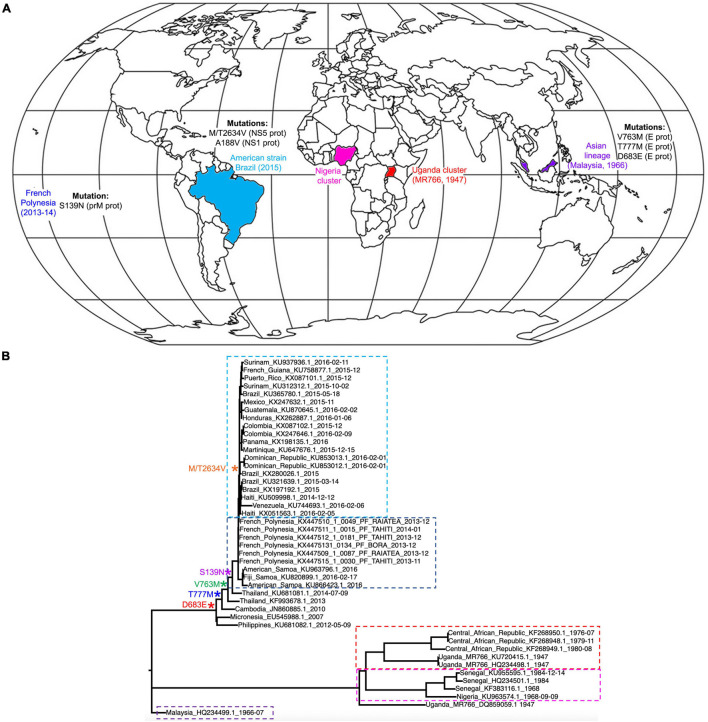
Lineage and global spread. **(A)** World distribution of ZIKV following its emergence in Uganda in 1947 and its diversion into African, Asian and American lineages. The map also indicates the main mutations that the different lineages have acquired in order to increase transmission and pathogenicity. **(B)** ZIKV phylogeny of African, Asian/Pacific and Latin American virus isolates, including mapping of amino acid substitutions cited in section “Mutations Affecting Viral Properties.” Selected sequences from Pettersson et al., 2016 were used to create a nucleotide phylogenetic tree under maximum likelihood using the IQTREE web server (Trifinopoulos et al., 2016). For clarity, Ugandan cluster isolates are boxed in red, Nigerian in pink, Malaysian in purple, French Polynesian in dark blue and American in light blue.

Following the emergence of ZIKV in Uganda in 1947, the virus eventually diverged into two main lineages: African and Asian/American (Figure 2A; Weaver et al., 2016). These differ from each other by approximately 3.5% at amino acid level (Willard et al., 2017). The African lineage is split into two groups: the Uganda cluster, which encompasses the original MR766 strain, and the Nigeria cluster that emerged following the westward expansion of the virus. The Asian lineage was derived from an African strain and following its initial identification in Malaysia in 1966 expanded throughout Southeast Asia, eventually causing a large outbreak in the French Polynesia in 2013–2014 (Cao-Lormeau et al., 2014). The contemporary American subclade evolved from this lineage, and showed the ability to rapidly transmit through South America’s immunologically naïve population (Weaver et al., 2016). Due to the close phylogenetic relationship of the American ZIKV subclade and strains isolated in Asia from 2007 onward, the studies discussed in this review that have used Asian strains should be considered relevant to American ZIKV. To allow ease of reading, the strains used in each study have been broadly referred to as “African,” “Asian,” or “American,” however, the reader can refer to Table 1 to identify specific strains that were used.

**TABLE 1:** ZIKV strains used in the studies discussed in this review.

**Lineage**	**Strain**	**References**
African	MR766	Dang et al., 2016
		Qian et al., 2016
		Zhang et al., 2016
		Anfasa et al., 2017
		Cortese et al., 2017
		Gabriel et al., 2017
		Sheridan et al., 2017
		Cortese et al., 2019
		Saade et al., 2020
		Yang et al., 2020
		Chen et al., 2021
		Han et al., 2021
	IbH30659	Chen et al., 2021
	Kedougou2011 Kedougou2015	Aubry et al., 2021
	976 Uganda	Anfasa et al., 2017
Asian	PF13/251013-18	Ghouzzi et al., 2016
		Anfasa et al., 2017
		Cortese et al., 2017
		Gabriel et al., 2017
		Gladwyn-Ng et al., 2018
		Cortese et al., 2019
		Saade et al., 2020
		Aubry et al., 2021
		Chen et al., 2021
	FSS13025	Zhang et al., 2016
		Sheridan et al., 2017
		Aubry et al., 2021
	SZ01/2016/China	Wu et al., 2016
		Han et al., 2021
	CPC-0740	Aubry et al., 2021
	SV0127-14	Aubry et al., 2021
	ZIKVNL00013	Anfasa et al., 2017
American	FB-GWUH-2016	Gabriel et al., 2017
	PRVABL59	Bardina et al., 2017
	PRVABC59	Clancy et al., 2018
		Yang et al., 2020
		Aubry et al., 2021
		Chen et al., 2021
	Suriname (Z1106033)	Saade et al., 2020

### Mutations Affecting Viral Properties

Multiple mutations differentiate the French Polynesia ZIKV strain from the African ZIKV strains (Figure 2B). These were acquired during the circulation of ZIKV through Asia, and some may be relevant to the changes that were seen in the properties of the virus. For example, the E protein acquired several mutations, including a D683E amino acid substitution in the receptor sequence. As the E protein is integral to receptor binding and host cell entry, changes in this protein could have affected properties such as viral tropism (Pettersson et al., 2016; Agrelli et al., 2019). Furthermore, Asian ZIKV strains acquired multiple mutations in the prM protein, five of which have been thought to significantly change the protein structure. However, the mechanistic implications of changes in the prM protein are currently unclear (Pettersson et al., 2016).

Only one mutation differentiates the French Polynesian strain from the preceding Asian ZIKV strains (Figure 2B): the S139N mutation in prM (Pettersson et al., 2016). As microcephaly and GBS had not been reported in association with ZIKV infection in any outbreaks prior to the French Polynesia epidemic, there is a possibility that this mutation contributed to the neurovirulence of ZIKV, which is further supported by the evidence that the S139N mutation increases infectivity of ZIKV in human NPCs (Yuan et al., 2017).

The mutation M/T2634V arose in the protein NS5 between the French Polynesia and South American epidemics (Pettersson et al., 2016). As the Latin American epidemic was associated with marginally higher rates of CZS than the French Polynesian, it is possible that this mutation also increased the neurovirulence of ZIKV (Pettersson et al., 2016). However, there is currently little experimental evidence to support this view, and increased CZS rates in the American epidemic could instead relate to other factors such as host genetics and previous flaviviral immunity.

Additionally, studies using mosquitoes and ZIKV infected mice have revealed that an A188V substitution in the NS1 protein appears to boost secretion of NS1 into the host blood, and that increased NS1 antigenaemia is linked to enhanced ZIKV infectivity in mosquitos. This leads to conjecture that the Asian strain mutated to become more transmissible in mosquitos, partly accounting for the high rates of infection that were seen in South America (Liu et al., 2017).

### Clinical Manifestations in Newborns: Congenital Zika Syndrome

Congenital Zika syndrome (CZS) is a unique set of disorders seen in an infant that has acquired ZIKV infection *in utero.* Perhaps the most well-known feature of CZS is microcephaly. However, the syndrome also includes: increased muscle tone that restricts movement, limited range of motion around the joints, and ocular damage (CDC, 2019). CZS is debilitating in many aspects, as the syndrome has associations with severe intellectual disability, cerebral palsy, and epilepsy (Abuelo, 2007).

It is worth noting that post-natal development of neurological defects following foetal ZIKV infection is also possible (Paul et al., 2018). This was confirmed by a study on 13 infants born in Brazil during the 2015–2016 epidemic, who were not diagnosed with CZS at birth but whose mothers had been exposed to ZIKV during pregnancy. Eleven of these infants developed microcephaly, some from within 5 months of life. The infants also presented with symptoms such as dysphagia, hypertonia and chorioretinal abnormalities (van der Linden et al., 2016). These observations corroborated with studies on mice, where pups born to dams that were exposed to a mild ZIKV infection during pregnancy exhibited motor and behavioral deficits, in addition to post-natal growth impediments, despite no evidence of microcephaly at birth (Paul et al., 2018). Therefore, neurological defects resulting from congenital ZIKV infection are not only limited to microcephaly at birth, and consequently the monitoring of infants who were born to ZIKV exposed mothers is crucial during the first few years of life (van der Linden et al., 2016).

### Cellular Mechanisms by Which *in utero* Zika Virus Infection Results in Microcephaly

**FIGURE 3:**
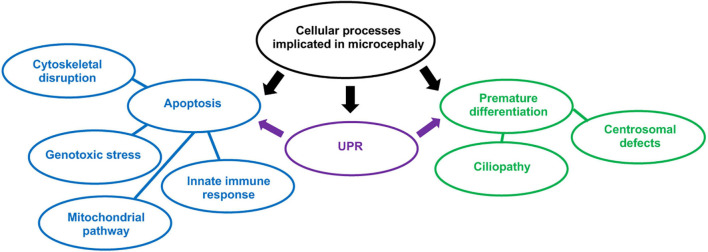
Cellular processes implicated in microcephaly resulting from *in utero* ZIKV infection. In blue, processes implicated in apoptosis and in green, NPCs premature differentiation. The UPR can trigger both apoptosis and premature differentiation of NPCs.

ZIKV is able to infect neural progenitor cells (NPCs) in the developing cerebral cortex when foetal infection occurs. These are progenitors that are capable of differentiating into neurons and glial cells (Martínez-Cerdeño and Noctor, 2018). Upon infection of NPCs, ZIKV can trigger either apoptosis or premature differentiation into mature neurons. This gives rise to the microcephaly phenotype, where the final number of neurons in the cerebral cortex is reduced. This review will discuss the cellular processes which could potentially cause ZIKV-induced apoptosis or premature differentiation (Figure 3). Notably, certain mechanisms that we will describe such as the activation of the unfolded protein response (UPR) can cause both apoptosis and differentiation. It is important to note that there is an imbalance of research regarding ZIKV-induced apoptosis and premature differentiation, as premature differentiation has only recently begun to be regarded as a possible cause of ZIKV-related microcephaly. Therefore, many of these studies may require further verification.

In addition, understanding which pathological mechanisms cause microcephaly will allow a judgement of which ZIKV strains are more likely to induce NPC apoptosis or differentiation, leading to a conclusion of whether a certain lineage is more prone to causing microcephaly.

## Apoptosis of Neural Progenitor Cells

### Zika Virus Infection of Neural Progenitor Cells at the Ventricular Zone

**FIGURE 4:**
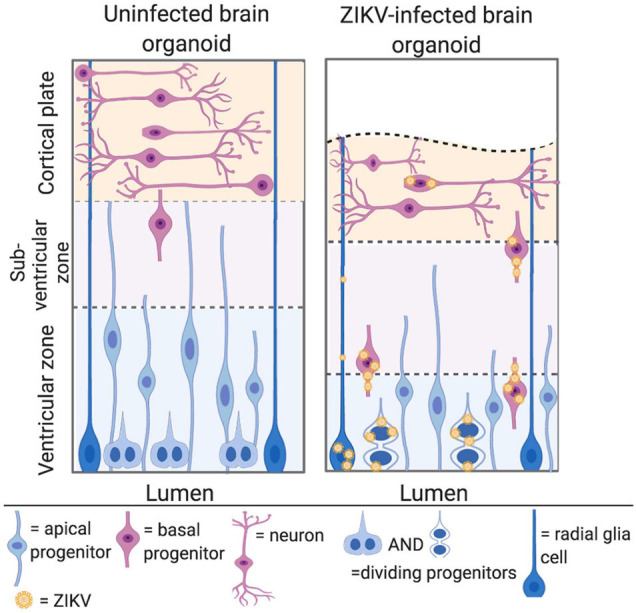
ZIKV can infect NPCs in the developing cerebral cortex. *Left panel:* cerebral organoids are organised into a ventricular zone, subventricular zone, and cortical plate. Apical NPCs are shown in blue and reside at the apical/luminal side of the organoid, where the ventricular zone is located. Basal progenitors are shown in pink, and divide at the subventricular zone, further from the apical surface. Neurones are shown in pink at the cortical plate at the basal side of the organoid. *Right panel:* ZIKV infected cerebral organoid. ZIKV virions are shown in yellow. The cortical plate is reduced in volume, owing to apoptosis and premature differentiation of NPCs. Asian ZIKV localises to the apical NPCs at the ventricular zone, whilst African ZIKV infects both the apical NPCs and neurons at the cortical plate (adapted from Gabriel et al., 2017, created with Biorender.com).

Studies have consistently shown that Asian/American and African ZIKV can infect NPCs in the developing cerebral cortex. Both African and Asian ZIKV strains infect around 60% of NPCs in a monolayer culture by 7 days post infection (Gabriel et al., 2017). The specific neurotropism of African and Asian ZIKV has been studied using cerebral organoids, which are derived from pluripotent stem cells and model the architecture of the human brain (Qian et al., 2016; Gabriel et al., 2017; Alves et al., 2018). The apical side of the organoid comprises a ventricular zone, where apical NPCs divide, whilst the basal side predominantly consists of neurons in the primitive cortical region (Figure 4, left) (Gabriel et al., 2017). Asian ZIKV has been shown to localise to apical NPCs at the ventricular zone, and whilst African ZIKV shares this tropism, it can also infect neurons in the primitive cortical region (Figure 4, right) (Qian et al., 2016; Gabriel et al., 2017).

Both African and Asian/American ZIKV strains cause apoptosis of NPCs. Ghouzzi et al. (2016) showed that infection of NPC cultures with Asian ZIKV induced nuclear pyknosis and increased levels of caspase-3, both of which are indicative of apoptosis. Interestingly, a significant number of apoptotic cells appeared to be uninfected, indicating that ZIKV potentially triggers a non-cell autonomous apoptotic mechanism. A comparison of an African and Asian strain showed that the African strain both infected a greater proportion of NPCs and caused greater levels of apoptosis (Zhang et al., 2016). This concurs with multiple studies that have demonstrated African strains to have a higher productivity and virulence in NPCs (Anfasa et al., 2017; Simonin et al., 2017). It is therefore possible that the African lineage is more neurovirulent than the Asian/American lineage.

### p53 Activation From Genotoxic Stress and the Innate Immune Response

RNA sequencing of NPC cultures has shown that targets of p53 are consistently upregulated following infection with Asian ZIKV (Ghouzzi et al., 2016; Zhang et al., 2016). Ghouzzi et al. (2016) confirmed that Asian ZIKV activates p53, as infected NPCs exhibited an increased accumulation of p53 in the nucleus, and immunoreactivity analysis with antiphospho-serine 15 (Ser15) p53 antibodies revealed that p53 had been activated via phosphorylation at the Ser15 residue. Furthermore, the activation of p53 correlated to the apoptosis of NPCs, as caspase-3-positive cells showed increased levels of phospho-Ser15 p53 immunoreactivity. Therefore, it is likely that the activation of p53 is integral to a mechanism by which Asian/American ZIKV strains cause the apoptosis of NPCs and consequent microcephaly.

In addition, Ghouzzi et al. (2016) checked the physiological relevance of these findings by comparing RNA sequencing profiles of ZIKV-infected NPCs with profiles of neural tissues from mouse models of genetic microcephaly, which have gene knockouts causing NPC apoptosis during development. These mouse models mirrored the upregulation of p53 targets, confirming the role of p53 in microcephaly.

There are multiple possibilities as to why ZIKV causes the p53 dependent apoptosis of NPCs:

(i) Genotoxic stress (stress due to DNA damage) as a consequence of viral infection. It has been shown that Asian ZIKV causes DNA damage, as phosphorylated H2AX histone levels were found to be increased in infected NPC cultures, indicating the presence of double stranded DNA breaks (Ghouzzi et al., 2016). Furthermore, it is known that DNA repair genes are downregulated following infection with Asian ZIKV (Zhang et al., 2016). DNA damage does not appear to be exclusive to NPCs with a high viral load, indicating that DNA damage is caused early on in the infection cycle (Ghouzzi et al., 2016). It will be important to test whether the same observations of p53 activation and genotoxic stress can be observed following infection of NPCs with African ZIKV strains, in order to draw a direct comparison between the two lineages.
(ii) Toll-like receptor 3 (TLR3) activation of the innate immune response may also trigger p53 dependent apoptosis (Ghouzzi et al., 2016). Activation of TLR3 results in the transcription of IFN-β, which operates via the JAK-STAT signalling pathway to stimulate the transcription of Interferon-Stimulated Response Element (ISRE) regulated genes, including p53 (Rivas et al., 2010). Therefore, TLR3 upregulation following ZIKV infection can be linked to p53 mediated apoptosis. Dang et al. (2016) verified this hypothesis by demonstrating that TLR3 is involved in the neuropathogenic properties of ZIKV. The application of a TLR3 agonist (i.e., polyl I:C) to cerebral organoids caused a shrinkage that mimicked the effects of infection with African ZIKV, and this could be reversed with a TLR3 antagonist (i.e., thiophenecarboxamidopropionate). TLR3 expression is temporally regulated in the developing cerebral cortex, and its expression is reduced as NPCs differentiate into mature neurones. Therefore, TLR3 induced apoptosis may explain why CZS is more likely to be an outcome of infection in the first trimester of pregnancy (Dang et al., 2016).

However, definitive conclusions regarding the involvement of TLR3 activation in American ZIKV neuropathogenesis will require studies specifically using Asian/American strains, as it should not be assumed that results from African ZIKV can be fully extrapolated to contemporary ZIKV strains.

### Mitochondrial Apoptosis Pathway

The process of apoptosis occurs by one of two broad mechanisms: the intrinsic or the extrinsic pathway. The intrinsic pathway, also known as the mitochondrial apoptosis pathway, is triggered by internal cellular stress, such as DNA damage. Subsequent activation of p53 causes the oligomerisation and activation of the pro-apoptotic proteins Bax and Bak in the outer mitochondrial membrane and the leakage of cytochrome C from the mitochondria to the cytoplasm. This triggers the activation of caspase 9, and subsequently caspases 3 and 7, leading to cellular destruction (Elmore, 2007). Both the African and Asian/American ZIKV lineages trigger the mitochondrial pathway of apoptosis (Yang et al., 2020; Han et al., 2021). Western blot analysis of infected ZIKV cells reveals activation of caspases 3, 7, and 9, which mediate the intrinsic pathway, but not caspase 8 which is integral to the extrinsic pathway (Yang et al., 2020). Furthermore, siRNA experiments to knockdown caspase 9 significantly prevented loss of cell viability 24 h after ZIKV infection (Han et al., 2021).

ZIKV NS4B protein activates Bax and thus triggers the mitochondrial pathway of apoptosis. Han et al. (2021) showed that Bax is integral to the mechanism that ZIKV infection induces apoptosis, as siRNA knockdown experiments silencing Bax significantly decreased loss of cell viability following ZIKV infection. This is likely a result of a direct interaction between NS4B and Bax, as NS4B was shown to localise to the mitochondria of infected cells. It has been proposed that NS4B localisation at the outer mitochondrial membrane can trigger the exposure of the N-terminal epitope of Bax, which subsequently destabilises the mitochondrial membrane (Han et al., 2021).

Mitochondrial fragmentation has been shown to precede caspase activation and apoptosis in infected ZIKV cells (Yang et al., 2020). This is because ZIKV infection causes a decrease in the levels of MFN2 protein, which is a mediator of mitochondrial fusion. This disturbs the balance in mitochondrial dynamics between fission and fusion, causing fragmentation. These effects have been directly implicated in ZIKV-mediated apoptosis, as treatment with the mitochondrial division inhibitor (Mdivi-1), which inhibits the process of mitochondrial fission, reverses the change in mitochondrial morphology, and prevents subsequent caspase 3/7 activation and cell death (Yang et al., 2020). These observations were made with both African and American ZIKV strains, indicating that mitochondrial fragmentation is relevant to the pathogenesis of both lineages.

### Cytoskeletal Disruption

During neural development, the cytoskeleton is involved in different processes that are fundamental to the generation and maturation of neurons, including but not limited to: (i) orientation of the mitotic spindle, (ii) interkinetic nuclear migration, (iii) microtubule organising centre centrosome activity, and (iv) assembly of primary cilia. The disruption of any of these processes impairs neurogenesis (Compagnucci et al., 2016).

Confocal microscopy of NPC cultures has shown that both African and Asian ZIKV strains induce dramatic rearrangements of the host cytoskeletal network (Cortese et al., 2017). ZIKV infected NPCs have a kidney shaped nucleus, with viral inclusion bodies containing viral non-structural (NS) proteins and dsRNA accumulated on the concave side. The microtubule network local to the perinuclear region collapses, with intermediate filaments and microtubules forming cytoskeletal cages around the viral inclusion bodies. Cytoskeletal cages are advantageous to the virus in two ways. Firstly, they are speculated to shield the highly immunogenic replication intermediate from innate immune recognition. Secondly, the enclosure of replication substrates and NS proteins is required for efficient genomic replication (Cortese et al., 2017).

Cytoskeletal modifications that occur following ZIKV infection are likely to contribute to NPC apoptosis and the resulting microcephaly phenotype (Cortese et al., 2017). This is potentially because intermediate filaments play a role in the inhibition of apoptosis by regulating the density of pro-apoptotic Fas receptors at the cell surface (Marceau et al., 2007; Cortese et al., 2017). Cells lacking the filament proteins keratin 8 and 18 have increased Fas receptors at the cell surface, and consequently increased activation of caspase 3 and apoptosis (Marceau et al., 2007). However, Cortese et al., 2017 used hepatocytes, therefore, further confirmation is needed that disruption of the NPC intermediate filament network following ZIKV infection induces apoptosis, and subsequent neurological defects.

Both African and Asian/American ZIKV strains cause disruption of the cytoskeletal network in order to enclose viral inclusion bodies within cytoskeletal cages. This potentially disrupts processes that are involved in neurogenesis, in addition to deregulating the inhibition of NPC apoptosis.

In addition, the role of the cytoskeleton in ZIKV neuropathogenesis will be further discussed in this review, in relation to centrosomal dysfunction and subsequent premature differentiation.

## Unfolded Protein Response Activation in Zika Virus-Infected Neural Progenitor Cells

The ultrastructural changes that occur during ZIKV infection cause ER stress, defined by the accumulation of misfolded proteins above a certain threshold (Alfano et al., 2019). This consequently triggers the unfolded protein response (UPR), a collection of signalling cascades that aims to restore protein folding homeostasis. If homeostasis is not achieved, the UPR becomes “terminal,” ultimately leading to apoptosis (Hetz and Papa, 2018). There is abundant evidence indicating that Asian/American ZIKV triggers activation of the UPR. NPCs infected with Asian ZIKV show increased levels of ER stress markers such as calnexin and calreticulin, and the upregulation of proteins involved in the PERK-eIF2α-ATF4 pathway, a signalling cascade of the UPR (Gladwyn-Ng et al., 2018). Similar results were drawn from the quantitative real-time polymerase chain reaction (qRT-PCR) of cortical tissue from ZIKV infected foetuses in the second trimester, confirming that UPR upregulation is physiologically relevant to human ZIKV infection (Gladwyn-Ng et al., 2018).

**FIGURE 5:**
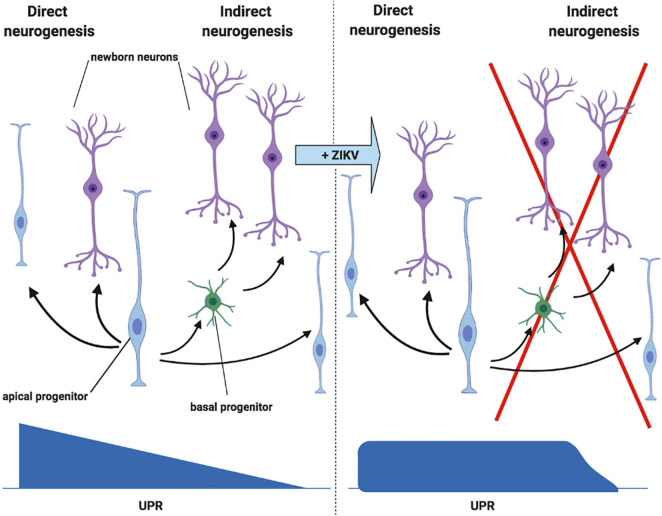
ZIKV infection deregulates the UPR during cortical neurogenesis. *Left panel:* initially, direct neurogenesis occurs, whereby each apical NPC gives rise to one neuron and another apical NPC. Indirect neurogenesis is then initiated later on in development, during which basal progenitors are produced. Basal progenitors divide symmetrically to either produce two further progenitors, or two neurons. Progressive decrease in UPR activity mediates the switch from direct to indirect neurogenesis. *Right panel:* the shift from direct to indirect neurogenesis is inhibited by ZIKV, which increases UPR activity following infection of the NPCs (adapted from Alfano et al., 2019, created with Biorender.com).

In order to understand the effect of UPR deregulation on cortical development, it is first necessary to understand the process of neurogenesis (Figure 5, left). Initially, apical NPCs divide symmetrically, rapidly expanding the progenitor pool. Asymmetric division (known as direct neurogenesis) also occurs, whereby each apical NPC gives rise to one neuron and another apical NPC. Indirect neurogenesis is initiated further on, during which basal progenitors are produced (Gladwyn-Ng et al., 2018; Alfano et al., 2019). Whilst apical progenitors divide in the ventricular zone, basal progenitors divide in the subventricular zone, further from the apical surface (Figure 4, left) (Arai and Taverna, 2017). Basal progenitors divide symmetrically to either produce two further progenitors, or mature neurons (Figure 5). Therefore, indirect neurogenesis is necessary to amplify the pool of NPCs and increase neuronal output (Gladwyn-Ng et al., 2018; Alfano et al., 2019). Newly generated neurons lose their apical process attachment to the ventricular surface and migrate basally (Noctor et al., 2004). The UPR is key to the temporal regulation of these processes, as a progressive decrease in UPR activity induces the shift toward indirect neurogenesis. Therefore, increased UPR activity as a consequence of ZIKV infection inhibits indirect neurogenesis and subsequently reduces neuronal output (Figure 5, right) (Alfano et al., 2019). Gladwyn-Ng et al. (2018) showed this by injecting ZIKV into embryonic mice brains and fate mapping the output of GFP electroporated NPCs. ZIKV infection diminished the production of NPCs, indicating that premature differentiation had occurred, and this was rectified by the co-administration of a UPR inhibitor with ZIKV.

In addition to reducing neuronal output from NPC progenitors, UPR activation also results in the apoptosis of the daughter neurons. Immunofluorescent microscopy of embryonic mice brains infected with ZIKV showed that the majority of apoptotic cells were neurons of the post-mitotic cortical region, as opposed to NPCs in the ventricular zone. Therefore, it has been theorised that the activation of the UPR limits the long term neuronal survival of NPC progeny (Gladwyn-Ng et al., 2018).

Overall, it is likely that the UPR activation following ZIKV infection of NPCs causes decreased neuronal output via the inhibition of indirect neurogenesis, and the apoptosis of progeny neurons. The corroboration of *in vitro* findings with those of the transplacental mouse model and *post mortem* tissue of ZIKV infected foetuses confirms that this mechanism is very likely to contribute to the neurological defects that follow foetal ZIKV infection. Although something similar is likely to occur with African ZIKV infection, these procedures should be repeated with an African ZIKV strain in order to allow a direct comparison of UPR mediated neuropathogenesis between the two lineages.

## Premature Differentiation of Neural Progenitor Cells

### Infected Neural Progenitor Cells Prematurely Differentiate

Recent studies have shown that ZIKV is also able to induce premature differentiation of NPCs into mature neurons (Gabriel et al., 2017; Saade et al., 2020). Notably, the levels of generated mature neurons did not accumulate and remained at a steady level, indicating that ZIKV is able to trigger differentiation of NPCs and then induce apoptosis of the newly generated neurons (Gabriel et al., 2017).

### Centrosomal Defects and the Mitotic Division Plane

The premature differentiation of NPCs induced by ZIKV infection has been linked to centrosomal dysfunction. ZIKV infection has been demonstrated to reduce the recruitment of key centrosomal proteins, such as CPAP (Gabriel et al., 2017). CPAP forms a layer around centrioles and interacts with pericentriolar material to form functional centrosomes, allowing the anchoring and recruitment of microtubules (Zheng et al., 2014). Many of the centrosomal proteins which are affected by ZIKV infection have also been implicated in autosomal recessive primary microcephaly (Bond et al., 2005; Gabriel et al., 2016). This validates the hypothesis that centrosomal dysfunction is physiologically relevant to CZS.

The inhibition of recruitment of centrosomal proteins causes a change in the mitotic division plane of ZIKV infected NPCs, resulting in premature differentiation (Figure 4, right). The division plane is horizontal during symmetric division, and a switch to a vertical division plane whereby NPCs are oriented toward the lumen of the neural tube reflects a shift to asymmetric division, and consequent differentiation (Gabriel et al., 2017). It has been shown using confocal microscopy that ZIKV infection causes the switching of the mitotic division plane. Infected cerebral organoids exhibited an increased proportion of NPCs in the ventricular zone with vertical division planes, with a resulting decreased NPC/neuron proportion. This occurs following infection with both Asian/American and African ZIKV strains, leading to the speculation that both lineages are capable of inducing premature differentiation by causing centrosome dysfunction (Gabriel et al., 2017).

### Ciliopathy and Delamination

**FIGURE 6:**
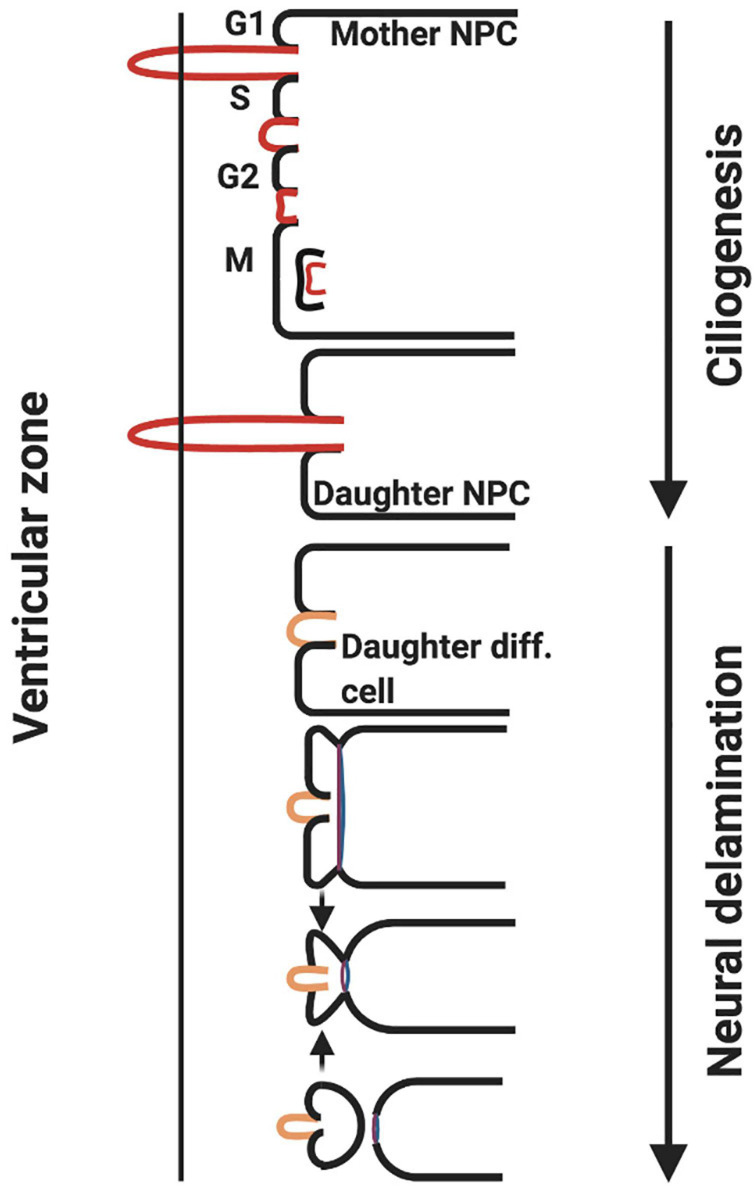
ZIKV causes ciliopathy and forces premature neurogenesis. During asymmetric division of apical NPCs, the daughter cell that reforms the primary cilia remains attached to the ventricular surface whereas the other daughter cell that does not undergo ciliogenesis delaminates from the ventricular surface and differentiates. ZIKV NS5 protein interacts with cellular proteins at the base of the primary cilia in NPCs causing an atypical ciliopathy and premature neuron delamination (adapted from Saade et al., 2020, created with Biorender.com).

Cilia length is crucial to the control of neurogenesis, as the primary cilium is required to maintain attachment of the NPC to the ventricular surface of the neural tube and thus prevent delamination (detachment). During asymmetric division, the daughter cell inheriting the mature centriole reforms the primary cilia, thus maintaining attachment to the ventricular surface and the status as an NPC. The other daughter cell will not reform a primary cilium, and will thus undergo delamination, basal migration and maturation into a neuron (Figure 6) (Gilmore and Walsh, 2013; Saade et al., 2020). Inheritance of the mature centriole is key to reforming the primary cilium, as it forms the core of the primary cilium basal body structure, and associates with Cep164 centrosomal protein to allow primary cilia assembly (Graser et al., 2007).

Infection of the developing foetal brain with ZIKV results in shorter cilia being reformed following NPC division. This is mediated specifically by the viral polymerase (NS5) (Saade et al., 2020). NPCs electroporated with NS5 and no other viral NS proteins exhibit significantly reduced cilia lengths. NS5 polymerase achieves this through localising to the base of primary cilia in NPCs and preventing the association of proteins such as Cep164, rootletin and BART, which are required for ciliogenesis (Graser et al., 2007; Saade et al., 2020). Shortened cilia will result in a loss of attachment of the NPC to the ventricular surface and subsequent differentiation into a neuron (Saade et al., 2020). In addition, there is another possible explanation for why shortened cilia drive premature differentiation: primary cilia are responsible for driving pro-proliferative signalling pathways, such as Wnt (Graser et al., 2007; Saade et al., 2020). The disruption of Wnt signalling has also been implicated in primary microcephaly, which is associated with mutations in the *ASPM* protein, a key signalling component of the Wnt pathway (Molenaar et al., 1996; Saade et al., 2020).

Saade et al. (2020) performed a comparative study between the African, the Asian and the American NS5 polymerase in order to decipher whether a lineage was more capable of inducing ciliopathy in NPCs. According to their studies all NS5 polymerases resulted in premature delamination from the ventricular surface.

## Zika Virus-Induced Brain Calcifications

CZS is associated with brain calcifications, which are the abnormal depositions of calcium phosphate crystals in neural tissue (CDC, 2019; Chen et al., 2021). Calcifications in the developing neural tissue are likely to contribute to the neurological symptoms seen in infants that have been infected *in utero* (Pool et al., 2019; Chen et al., 2021).

Chen et al. (2021) showed that Asian ZIKV can infect foetal pericytes in the brain to subvert the osteogenic signalling and trigger calcification. Pericytes are cells found in capillary walls throughout the body. In the brain, they are important for processes such as vascular development, regulation of cerebral blood flow, and maintaining the integrity of the blood brain barrier (Brown et al., 2019). ZIKV infection of pericytes is likely integral to the mechanisms of brain calcification, as analysis of ZIKV infected foetal brains in the 2015–2016 epidemic revealed calcifications surrounded by ZIKV-RNA positive pericytes (Chen et al., 2021). Upon infection of foetal pericytes, ZIKV has been shown to interfere with the BMP2-SMAD pathway which is usually associated with physiological bone formation (Rosen, 2009; Chen et al., 2021). In normal osteogenic signalling, pro-BMP2 is cleaved by furin-type proteases to its active form and secreted from the cell. Mature BMP2 then interacts with BMP I/II receptor to trigger the phosphorylation of SMAD1/5/9, which subsequently translocates to the nucleus to induce the expression of osteogenic master transcription factor Runt-related transcription factor 2 (RUNX2). Downstream osteogenic and effector genes are then expressed and cause calcification (Lee et al., 2015; Chen et al., 2021). Infection of foetal pericytes with Asian ZIKV led to increased levels of BMP2 in the supernatant, increased expression of key osteogenic genes such as *RUNX2*, and calcification (Chen et al., 2021).

ZIKV infection subverts the BMP2-SMAD signalling pathway in pericytes through the NS3 protease. The expression of ZIKV NS3 led to increased mRNA levels of *BMP2, RUNX2*, and other osteogenic genes. These effects were absent upon expression of a mutant NS3 lacking the protease capability (Chen et al., 2021). It is likely that the NS3 protease acts by cleaving pro-BMP2 to its active form, which is then secreted from pericytes and induces osteogenic signalling to trigger calcifications (Chen et al., 2021).

It is worth noting that in this study, the utilised African ZIKV strain failed to induce BMP2 and osteogenic gene upregulation in foetal pericytes. It is speculated that African ZIKV is unable to cause calcifications of the foetal brain due to its possible higher virulence, which prevents sustained upregulation of BMP2 and osteogenic genes (Chen et al., 2021).

## Congenital Zika Virus Syndrome in Africa

Many studies have found African ZIKV strains to be significantly more productive and virulent in a range of relevant cells, including NPC cultures (Zhang et al., 2016; Anfasa et al., 2017; Simonin et al., 2017). It is therefore possible that rather than causing microcephaly, infection with African ZIKV during pregnancy results in complete foetal loss (Sheridan et al., 2017; Aubry et al., 2021). This would account for the fact that microcephaly was not reported in association with ZIKV infection during its circulation in Africa. Intraplacental injection of African ZIKV into immunocompetent mouse embryos at embryonic day 10.5 (E10.5) caused complete foetal death before E18.5. In contrast, infection with the Asian ZIKV did not cause foetal loss, but instead microcephaly and ventriculomegaly, with reduced cortical thickness and reduction in head weight (Aubry et al., 2021). It is possible that ZIKV became attenuated prior to the 2015–2016 epidemic in Latin America, which meant that foetuses survived *in utero* infection, and thus the neuropathogenic nature of the virus could become apparent (Aubry et al., 2021).

On the other hand, it is still possible that African ZIKV has the potential to cause microcephaly, but this went undiscovered during its circulation due to inadequate public health infrastructure in Africa. A lack of funding in public health has made surveillance difficult, and this is unlikely to change without conclusive evidence that African ZIKV causes CZS (Wetsman, 2017). Furthermore, due to the presence of other circulating pathogens in Africa that cause congenital birth defects (TORCH infections), it is likely that any cases of microcephaly would have been attributed to these, rather than ZIKV (Wetsman, 2017).

In addition to a lack of public health surveillance, there are experimental challenges with African ZIKV that prevent definitive conclusions being drawn on whether it can cause CZS. Studies on the African lineage predominantly use the strain MR766, which was the first isolated ZIKV strain from 1947. In order to grow the virus for continued experimental studies, the strain was passaged approximately 150 times through mice brains raising adaptative mutations to optimise growth in neural tissue (Wetsman, 2017). As a result, experimental data based on the use of the MR766 ZIKV strain cannot be used to reach definitive conclusions about the virulence of African ZIKV (Wetsman, 2017).

## The Effect of Dengue Immunity on Zika Virus Infection

There are other theories that may explain the high rates of microcephaly seen in the American ZIKV epidemic, one of which is the effect of pre-existing DENV immunity on the clinical outcome of ZIKV infection. It is already well documented that previous infection with one DENV serotype can enhance infection with another serotype, in a phenomenon called “antibody dependent enhancement” (ADE) (Flipse et al., 2016). As ZIKV and DENV are closely related flaviviruses with a close antigenic relationship and overlapping geography in South America, it may be that the principle of ADE can be extended to ZIKV infection. However, both *in vitro* and *in vivo* studies have thus far been conflicting in establishing whether DENV immunity protects against, or enhances ZIKV infection (Bardina et al., 2017; Rodriguez-Barraquer et al., 2019).

On one hand, Stat2^–/–^ mice given plasma from DENV infected donors and infected with Asian ZIKV showed a decreased survival rate by day 8, when compared to mice that received control plasma (21.4 and 93.3% respectively). Additionally, these mice exhibited enhanced weight loss, and increased neurological symptoms such as total body paralysis. This was reflected by an increased ZIKV RNA level and viraemia on day 3 of infection in comparison to controls. Therefore, this model gives rise to the speculation that cross reaction with DENV allows ZIKV infection to achieve a higher viral titre in the host and cause a more severe clinical outcome (Bardina et al., 2017). However, data taken from Brazilian communities exposed to ZIKV in the 2015 epidemic found that individuals with prior immunity to DENV had protection against ZIKV infection. Serological studies were used to identify IgG raised to the NS1 proteins of DENV and ZIKV, and those in the highest and middle tertile of DENV NS1 IgG showed a respective 38 and 44% reduction in the risk of obtaining ZIKV infection during the 2015 epidemic, in addition to a better prognosis upon infection (Rodriguez-Barraquer et al., 2019). This study was further verified by serological data collected from children aged 2–14 years old in Nicaragua, which when processed in a regression model showed that prior DENV infection was inversely related to the presentation of symptoms following infection with ZIKV (Gordon et al., 2019). However, this protection against clinical symptoms did not extend to the occurrence of CZS, as the rate of abnormal birth outcome amongst DENV IgG positive women was not significantly different to that of IgG negative women (Halai et al., 2017). Therefore, whilst there is vast conflicting evidence regarding whether DENV immunity is protective against ZIKV, or enhances the severity of infection, it appears unlikely that the prevalence of microcephaly during the 2015 epidemic can be attributed to DENV cross reactivity.

## Social Impact and Climate Change

The 2015 ZIKV epidemic had a devastating social impact on Latin American women and highlighted fundamental flaws in the reproductive health care systems of affected countries. Despite recommendations from health authorities to postpone pregnancies for up to two years, many women were deprived of easy access to long term contraceptives or safe abortive procedures due to the criminalisation of abortion in many Latin American countries (HRW, 2017) (Valente, 2017). This led to a surge of women undergoing illegal abortive procedures that involved dangerous methods such as caustic acid, resulting in increased maternal mortality (HRW, 2017).

Furthermore, the study of arboviruses (arthropod-borne viruses) is becoming increasingly critical due to climate change, with more regions becoming habitable for mosquitoes. Whilst DENV cases in particular have notably increased in Europe and the US, current models of viral transmission by *Aedes aegypti* and *Aedes albopictus* predict that as climate change progresses, there will be an growing cases of both ZIKV and DENV in Europe and higher altitude areas of Africa and South America (Ryan et al., 2019). The introduction of arboviruses into Europe is of great concern, following the severe impact that ZIKV had when introduced into immunologically naïve populations in America during the 2015–2016 epidemic (Ryan et al., 2019).

## Discussion and Conclusion

This review discusses that the neuropathogenic effects of foetal ZIKV infection cannot be attributed to a single mechanism, but rather the cumulative effect of multiple pathways that either result in the premature differentiation or apoptosis of NPCs in the developing cerebral cortex. In addition, as both lineages of ZIKV appear able to employ many of these pathways, it seems likely that African ZIKV is at least as neurovirulent as the Asian lineage. However, there have been some limitations to the research of ZIKV neuropathogenesis thus far.

For example, studies on ZIKV neuropathogenesis have been limited due to the absence of physiologically relevant *in vitro* and *in vivo* models that are able to accurately recapitulate foetal human ZIKV infection. The use of organoids in some studies has allowed a demonstration of the effect of ZIKV infection on the cerebral architecture that would not have been possible with NPC cultures. However, organoids still lack some of the more nuanced details of the developing foetal brain, such as an immune or vascular system (Qian et al., 2017).

In terms of animal models, non-human primates (NHP) are an excellent model for CZS research due to their genetic closeness to humans (e.g., placental organisation and long gestation periods). In addition, subcutaneous inoculation of ZIKV results in productive vertical transmission to the offspring similar to humans. The most utilised NHPs include olive baboons, pigtail macaques, rhesus macaques and marmosets (Narasimhan et al., 2020). However, studies using NHP models are arduous, lengthy, and very expensive.

Many studies have utilised the transplacental mouse model (Miner et al., 2016; Jagger et al., 2017; Jaeger et al., 2019), which also has some limitations. For example, it is not possible to experimentally infect these mice early in gestation before E9, as this results in pregnancy loss. However, it is debatable whether it is necessary to infect mice earlier than E10.5, which is the time that neurogenesis commences in the dorsal cerebral cortex (Mukhtar and Taylor, 2018). Furthermore, although the microcephaly phenotype tends to mirror that of a human infection with enlarged cerebral ventricles (ventriculomegaly) (Jagger et al., 2017; Jaeger et al., 2019), in some cases, ZIKV infection produces embryonic death before E18.5 (Aubry et al., 2021) or infected mice pups show shrunken ventricles instead (Wu et al., 2016). This seems to be strain-associated, with African ZIKV being more prone to causing embryonic loss.

It is also important to note that mice are not generally permissive to ZIKV infection, so it is necessary to hinder the type I IFN signalling system in order to allow ZIKV replication, often by genetic modification or chemical manipulation (i.e., type I IFN receptor specific blocking antibody) (Miner et al., 2016). However, some studies have shown successful vertical transmission in immunocompetent mice where high doses of virus (e.g., 10^10^ to 10^12^ PFU/mL), unconventional routes of inoculation (e.g., intravaginal inoculation), or humanised mice (i.e., a humanised STAT2 mouse strain exhibited vertical transmission of a mouse-adapted strain after subcutaneous inoculation at E6.5) have been used, as reviewed in Narasimhan et al., 2020.

As an alternative to mouse models, a non-typical and inexpensive *in vivo* model of vertical ZIKV infection is the chick embryo. Chick embryos can be infected with ZIKV very early in gestation at the time of neurogenesis onset, mimicking the 1st trimester foetal infection that appears to result in the most severe pathology in human infection. Chick embryos are able to recapitulate the human foetal microcephaly phenotype (ventriculomegaly, stunted growth of the CNS and gross microcephaly) (Goodfellow et al., 2016; Thawani et al., 2018); and are fully permissive to ZIKV infection without genetic modification.

It is important to note that many of the studies discussed in this review only used one ZIKV strain (see Table 1). This was generally either due to the assumption that the properties of both ZIKV lineages can be considered similar enough to extrapolate findings from one to the other, or due to a disregard of the possibility that African ZIKV may also be neuropathogenic and thus worth investigation.

Therefore, in order to decipher the phenotypic differences between the two lineages of ZIKV, it will be crucial to repeat studies using strains from both lineages, whilst using different *in vitro* and *in vivo* models.

## Author Contributions

EK and NI conceived the project and wrote the manuscript. Both authors contributed to the article and approved the submitted version.

## Conflict of Interest

The authors declare that the research was conducted in the absence of any commercial or financial relationships that could be construed as a potential conflict of interest.

## Publisher’s Note

All claims expressed in this article are solely those of the authors and do not necessarily represent those of their affiliated organizations, or those of the publisher, the editors and the reviewers. Any product that may be evaluated in this article, or claim that may be made by its manufacturer, is not guaranteed or endorsed by the publisher.
